# Impact of Light and Dark Treatment on Phenylpropanoid Pathway Genes, Primary and Secondary Metabolites in *Agastache rugosa* Transgenic Hairy Root Cultures by Overexpressing *Arabidopsis* Transcription Factor *AtMYB12*

**DOI:** 10.3390/life13041042

**Published:** 2023-04-19

**Authors:** Thi Minh Hanh Do, Minsol Choi, Jae Kwang Kim, Ye Jin Kim, Chanung Park, Chang Ha Park, Nam Il Park, Changsoo Kim, Ramaraj Sathasivam, Sang Un Park

**Affiliations:** 1 Department of Smart Agriculture Systems, Chungnam National University, 99 Daehak-ro, Yuseong-gu, Daejeon 34134, Republic of Korea; 2 Division of Life Sciences and Convergence Research Center for Insect Vectors, College of Life Sciences and Bioengineering, Incheon National University, Yeonsu-gu, Incheon 22012, Republic of Korea; 3 Department of Crop Science, Chungnam National University, 99 Daehak-ro, Yuseong-gu, Daejeon 34134, Republic of Korea; 4 Department of Biological Sciences, Keimyung University, Dalgubeol-daero 1095, Dalseo-gu, Daegu 42601, Republic of Korea; 5 Division of Plant Science, Gangneung-Wonju National University, 7 Jukheon-gil, Gangneung 25457, Republic of Korea

**Keywords:** *Agastache rugosa*, *AtMYB12*, metabolic profiling, primary metabolites, secondary metabolites

## Abstract

*Agastache rugosa*, otherwise called Korean mint, has a wide range of medicinal benefits. In addition, it is a rich source of several medicinally valuable compounds such as acacetin, tilianin, and some phenolic compounds. The present study aimed to investigate how the Tartary buckwheat transcription factor *AtMYB12* increased the primary and secondary metabolites in Korean mint hairy roots cultured under light and dark conditions. A total of 50 metabolites were detected by using high-performance liquid chromatography (HPLC) and gas chromatography–time-of-flight mass spectrometry (GC-TOFMS). The result showed that the *AtMYB12* transcription factor upregulated the phenylpropanoid biosynthesis pathway genes, which leads to the highest accumulation of primary and secondary metabolites in the *AtMYB12*-overexpressing hairy root lines (transgenic) than that of the GUS-overexpressing hairy root line (control) when grown under the light and dark conditions. However, when the transgenic hairy root lines were grown under dark conditions, the phenolic and flavone content was not significantly different from that of the control hairy root lines. Similarly, the heat map and hierarchical clustering analysis (HCA) result showed that most of the metabolites were significantly abundant in the transgenic hairy root cultures grown under light conditions. Principal component analysis (PCA) and partial least-squares discriminant analysis (PLS-DA) showed that the identified metabolites were separated far based on the primary and secondary metabolite contents present in the control and transgenic hairy root lines grown under light and dark conditions. Metabolic pathway analysis of the detected metabolites showed 54 pathways were identified, among these 30 were found to be affected. From these results, the *AtMYB12* transcription factor activity might be light-responsive in the transgenic hairy root cultures, triggering the activation of the primary and secondary metabolic pathways in Korean mint.

## 1. Introduction

Phenylpropanoids are a group of diverse natural compounds derived through a variety of enzymatic reactions from precursors like primary metabolites, phenylalanine, or tyrosine amino acids [1]. Phenylpropanoids played an important role in plants when responding to biotic and abiotic stimuli. In particular, they are indicators of plant responses to different levels of light intensity or mineral treatment [2]. According to the carbon skeleton, phenylpropanoids have been divided into six main subclasses including flavanones, flavones, isoflavones, flavonols, and anthocyanidins. Along with advantages for plant defences, phenylpropanoid compounds could play an essential role in improving human health [3]. They possess many biological activities such as antitumor [4], antibacterial [5,6], antioxidant [7,8], antiviral [9], anti-inflammatory [7,10], and neuroprotective activities [11].

Korean mint is a perennial medicinal plant and it is mostly found in East Asian countries such as China, Korea, and Vietnam. It has been reported that the plant has many medical applications such as anti-bacterial [12,13], antifungal [14], antiviral [15], and anti-inflammatory [10] activities as well as treating inflammatory gastric diseases [16] and preventing bone loss [17]. Korean mint consists of a diversity of secondary metabolites, including two main metabolic classes—phenylpropanoids and terpenoids. Among them, there are many non-volatile compounds such as phenolic compounds, several flavonols such as quercetin, and flavone glycosides such as acacetin, and tilianin [18]. Thus, this plant has been widely used in the pharmacology industry as a source of natural compounds for human disease treatment.

One of the strategies for increasing the synthesis of phenolic compounds is the overexpression of regulatory genes for inducing biosynthesis pathways. In previous studies, it has been reported that the MYB transcription factor families are involved in secondary metabolites [19,20]. For instance, *AtMYB003*, *AtMYB007*, *AtMYB032*, *AmMYB305*, and *AmMYB340* have functions in phenylpropanoid metabolism [21,22], whereas *AtMYB075*, *AtMYB090*, *AtMYB113*, and *AtMYB114* regulate anthocyanin accumulation [23]. In association with the MYB transcription factor family, *MYB12* regulates flavonoid biosynthesis by activating the transcription of chalcone synthase and flavonol synthase [24]. *MYB12* is reported as a transcription factor that controls flavonol accumulation in several plants such as *Solanum lycopersicum* (tomato), *Malus crabapple* (apple), *Malus sieversii* (apple), and Brassicaceae [25,26,27,28]. Hairy root cultures can produce a higher content of plant-derived natural secondary metabolites than that of the native host plant [29]. Hence, few studies have been carried out by transforming the *MYB12* transcription factor to hairy root cultures of *Fagopyrum esculentum* (common buckwheat) [30] and *Astragalus membranaceus* (Mongolian milkvetch) [31] for enhancing secondary metabolite contents. *AtMYB12* was isolated from *Arabidopsis thaliana* (thale cress) and played an important role in improving flavonoid accumulation [32]. However, to date, the effect of the *AtMYB12* gene on flavonoid accumulation in Korean mint hairy roots has not yet been well studied.

Light plays an essential role in plant physiology, as well as the modulation of gene expression and secondary metabolite biosynthesis [33]. In previous studies, it has been reported that light not only affects the hairy root growth of *Artemisia annua* (annual wormwood) [34] and *Agastache foeniculum* (anise hyssop) [35] but also increases the accumulation of phenolics content in *Daucus carota* (carrot) hairy root cultures [36] and *F. tataricum* (Tartary buckwheat) [37,38]. Among *MYB* family members, light can induce the *FtMYB116* transcription factor, which enhances the accumulation of rutin in Tartary buckwheat [39], or it can modulate flavonol content in transgenic tobacco by *AtMYB111* expression [40]. A previous study reported that light enhances the flavones and rosmarinic acid content in the seedlings of Korean mint [33]. To date, no studies have been carried out on the impact of light and dark on the overexpression of *AtMYB12* in Korean mint hairy roots.

This study carried out so that the transgenic hairy lines were developed by transferring the *AtMYB12* transcription factor into Korean mint explants via *Agrobacterium rhizogenes* [41,42]. To screen the highest *AtMYB12*-overexpressed hairy root lines, quantitative real-time (qRT) PCR was performed. The hairy root cultures that showed the highest *AtMYB12* expression were selected, and they were grown under light and dark conditions for 4 weeks. The primary and secondary metabolic compounds were analysed by using HPLC and GC-TOFMS. In addition, we analysed the expression level of phenylpropanoid and flavonoid pathway genes such as *ArPAL* (phenylalanine ammonia-lyase), *ArC4H* (cinnamate 4-hydroxylase), *Ar4CL* (4-coumarate: CoA ligase), *ArCHS* (chalcone synthase), *ArCHI* (chalcone isomerase) by qRT-PCR (Figure S1). This research could be helpful for the researchers to enhance the primary and secondary metabolites in Korean mint hairy root cultures by using a genetic engineering approach.

## 2. Results

### 2.1. AtMYB12-Overexpression Level in Transgenic Hairy Roots

The expression level of *AtMYB12* was analysed in the GUS-control and transgenic hairy root lines grown under dark conditions by using qRT-PCR, and the actin gene was used as an internal control. A total of 15 transgenic hairy root lines were developed, and all those lines were subjected to qRT-PCR analysis to evaluate the relative *AtMYB12* gene expression level by using a gene-specific primer. The results showed that the *AtMYB12* gene was highly upregulated in the transgenic line with the relative level ranging from 14 to 181-fold changes compared to the control line (Figure S2). Among the 15 transgenic lines, only three hairy root lines (M-3, M-4, and M-9) showed the highest level of *AtMYB12* overexpression with 113, 125, and 181-fold changes compared to control line, respectively. These three lines were selected for the HPLC analysis of phenolic compounds.

### 2.2. Total Phenolic Compounds in AtMYB12 Transgenic Hairy Roots

The selected three highest transgenic and control hairy root lines were subjected to HPLC analysis to determine the total phenolic content (Table S1). The total phenolic content ranged from 957.5 to 1203 µg/g dry weight (DW). Among the three transgenic lines, M-9 showed the highest total phenolic content, which is 1.25 times higher than the control hairy root line, followed by M-4, and M-3 lines, which were 1.16- and 1.05 times higher, respectively. This supports the result of *AtMYB12* gene expression, in which M-9 showed the highest gene expression. Hence, the M-9 transgenic line was chosen for further experiments.

### 2.3. Impact of Dark Treatment on Phenylpropanoid Pathway Genes Expression in Control and Transgenic Hairy Roots of Korean Mint

**Figure 1:**
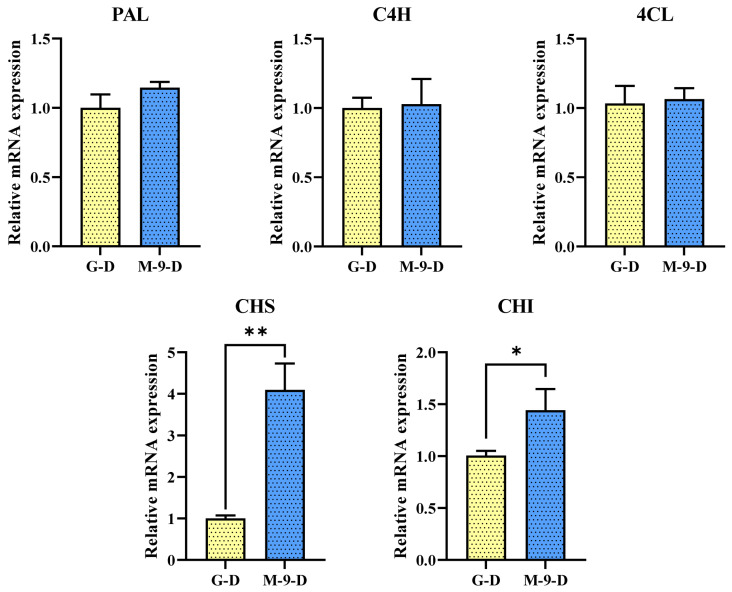
The relative gene expression level of phenylpropanoid biosynthetic pathway genes in Korean mint transgenic hairy root lines grown under dark conditions. Asterisks described statistical significance (* *p* < 0.05, ** *p* < 0.01). G-D: GUS-overexpressing hairy root line grown under dark conditions (control line); M-9-D: AtMYB12-overexpressing hairy root line grown under dark conditions (transgenic line).

To understand the regulatory effect of the *AtMYB12* transcription factor on the M-9 transgenic line, qRT-PCR was carried out to analyse the expression of phenylpropanoid pathway genes. The results showed that all five phenylpropanoid biosynthesis genes (*ArPAL*, *ArC4H*, *Ar4CL*, *ArCHS*, *ArCHI*) had a higher level of expression in the transgenic line than that of the control line when exposed to the dark condition. Among them, the expression of *ArCHS* in the transgenic line is the highest, which is 4.1-fold higher than the control line, followed by the *ArCHI* gene which was 1.4 times higher in comparison with the control line. Meanwhile, *ArPAL*, *ArC4H*, and *Ar4CL* did not show any significant differences between the control and transgenic line (Figure 1).

### 2.4. Impact of Dark Treatment on Phenolic and Flavones Content in Control and Transgenic Hairy Roots of Korean Mint

**Figure 2:**
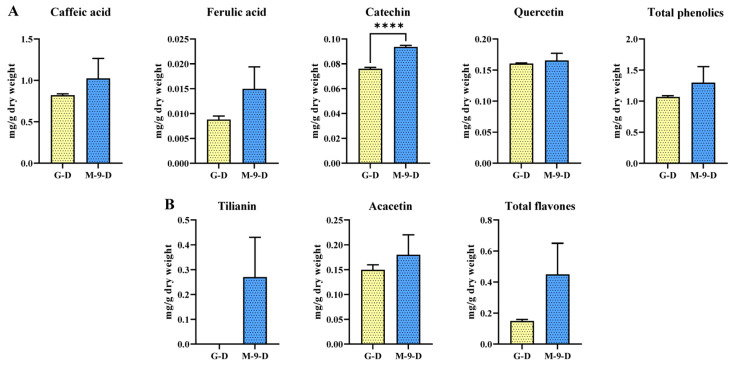
(**A**) Phenolic and (**B**) flavone contents in Korean mint hairy root lines grown under dark conditions. G-D: GUS-overexpressing hairy root line grown under dark conditions (control line); M-9-D: AtMYB12-overexpressing hairy root line grown under dark conditions (transgenic line). Asterisks described statistical significance (**** *p* < 0.0001).

The production of phenolics and flavones in Korean mint hairy root cultures (control and transgenic line) grown under dark conditions was analysed using HPLC. Four individual phenolic compounds (caffeic acid, ferulic acid, catechin, and quercetin) were detected in the control and transgenic lines (Figure 2A). The total phenolic contents showed a slightly distinctive accumulation between the control and transgenic hairy root line grown under dark conditions. The result showed that when exposed to dark conditions, the total phenolic content of the M-9 line was 1298.4 µg/g DW which was 1.21 times higher than that of the control line. Among the four quantified phenolic compounds, caffeic acid accounted for the majority, which was 821.8 and 1024.4 µg/g DW in the control and transgenic hairy root lines, respectively. From the result in Figure 2A, only the level of catechin showed a significant increase in the transgenic hairy root cultures, which was 1.23 times higher than in the control hairy root line. The remaining individual phenolic compounds did not show significant differences between the control and transgenic hairy root lines. This finding supported the phenylpropanoid gene expression results of Korean mint hairy root grown under dark conditions.

The flavone accumulation in control and transgenic hairy root lines grown under dark conditions was quantified by HPLC. The total flavone content of the M-9 line was 300 µg/g DW which was three times higher than the control line when exposed to the dark condition. Interestingly, tilianin was only detected in the transgenic hairy root line (270 µg/g DW) (Figure 2B), whereas acacetin was detected in both two lines, and the content in the control line was 1.2 times lower than the transgenic line grown under dark conditions.

### 2.5. Impact of Light Treatment on Phenylpropanoid Pathway Genes Expression in Control and Transgenic Hairy Roots of Korean Mint

**Figure 3:**
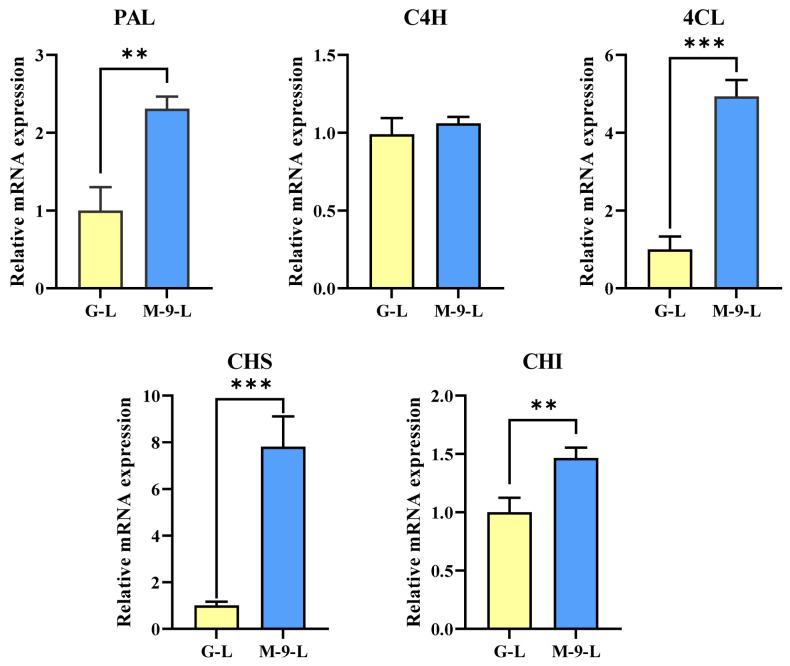
The relative gene expression level of phenylpropanoid biosynthetic pathway genes in Korean mint transgenic hairy root lines grown under light conditions. Asterisks described statistical significance (** *p* < 0.01; *** *p* < 0.001). G-L: GUS-overexpressing hairy root line grown under light conditions (control line); M-9-L: AtMYB12-overexpressing hairy root line grown under light conditions (transgenic line).

As shown in Figure 3, five phenylpropanoid pathway genes of Korean mint hairy root grown under light conditions were quantified by using qRT-PCR. Except for *ArC4H*, all other genes showed significantly differential expression between the control and transgenic hairy root lines. In particular, the transcript level of the *ArCHS* gene notably increased, with a 7.88-fold higher change in the transgenic hairy root culture lines than that of the control lines when exposed to continuous light. Similarly, the expression levels of *ArPAL*, *Ar4CL*, and *ArCHI* were significantly up-regulated in the transgenic line, which was 2.17-, 4.94-, and 1.47-fold higher than that of the control line grown under light conditions.

### 2.6. Impact of Light Treatment on Phenolic and Flavones Content in Control and Transgenic Hairy Roots of Korean Mint

**Figure 4:**
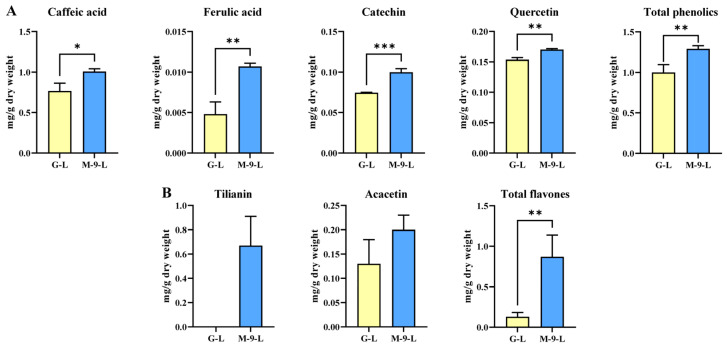
(**A**) Phenolic and (**B**) flavone contents in Korean mint hairy root lines grown under light conditions. G-L: GUS-overexpressing hairy root line grown under light conditions (control line); M-9-L: AtMYB12-overexpressing hairy root line grown under light conditions (transgenic line). Asterisks described statistical significance (* *p* < 0.05; ** *p* < 0.01, *** *p* < 0.001).

To determine the effect of the *AtMYB12* transcription factor on Korean mint hairy root, the accumulation of phenolics and flavones compounds in control and transgenic lines grown under light conditions was examined using HPLC. The results demonstrated that there was a remarkable increase in the phenolic content in the transgenic hairy root line compared to the control line (Figure 4A). The total phenolic content of the control line was 996.7 µg/g DW which was 1.3-times lower than the transgenic line (1295.7 µg/g DW). Four individual phenolic compounds were detected in Korean mint hairy root cultures, and all the phenolic compounds showed significant differences between the control and transgenic hairy root line grown under light conditions. In detail, the caffeic acid contents were the highest and were 767.1 and 1008.3 µg/g DW in the control and transgenic hairy root lines, respectively. In addition, the quercetin content was increased significantly in the transgenic line (170.3 µg/g DW), which was 1.13 times higher than the control line grown under light conditions. Furthermore, the content of catechin was also noticeably increased in the transgenic line grown in continuous light, which was 1.34-fold higher than the control hairy root line. Moreover, the ferulic acid content was significantly increased in the transgenic line, which was 2.23 times higher than that of the control line.

Similar to the phenolic compounds, the flavone content in the Korean mint hairy root cultures also showed a significant difference between the control and transgenic line when cultured under light conditions. Interestingly, tilianin was not detected in the Korean mint control hairy root line (Figure 4B), whereas it greatly increased in the transgenic line (670 µg/g DW). The total flavone content of the transgenic line increased dramatically, which was 6.7-times higher than the control line. In addition, when compared to the dark conditions, the light-exposed transgenic hairy root line showed a tremendous increase in the tilianin content (670 µg/g DW), which was 2.48-times higher than the dark conditions. Moreover, acacetin content in the transgenic hairy root line was 1.54-fold higher than the control in continuously lighted conditions. The phenolic and flavone content of the transgenic hairy root cultures was consistent with the results of phenylpropanoid pathway gene expression of Korean mint under light conditions.

### 2.7. Metabolic Profiling of Identified Metabolites from Control and Transgenic Hairy Root Lines

**Figure 5:**
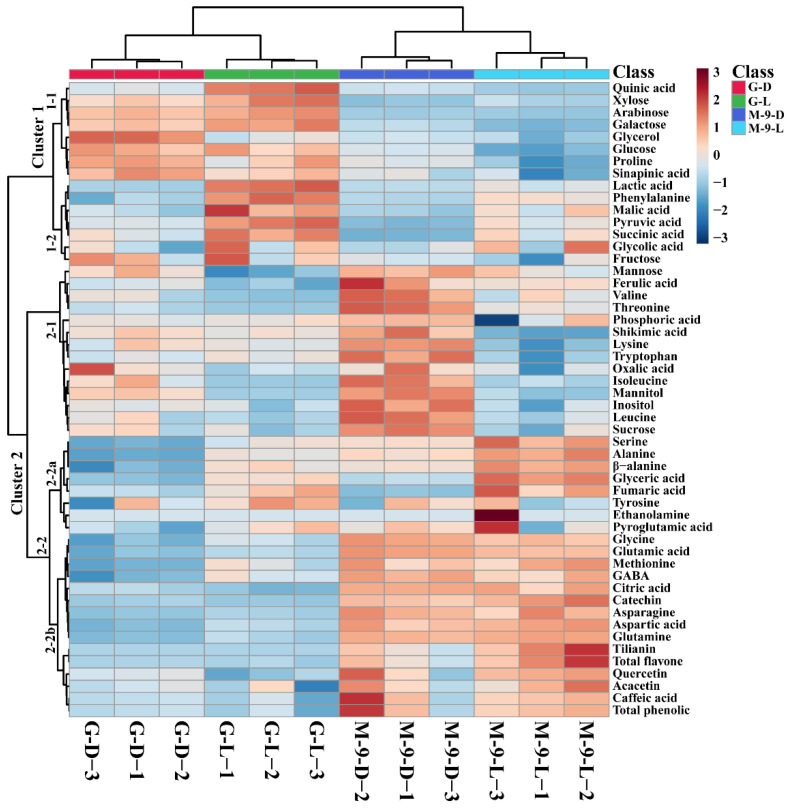
Heatmap showing variations in the several metabolite levels in control and transgenic Korean mint hairy root lines grown under light and dark conditions; the metabolites’ increases and declines are depicted in red and blue, respectively. G-D: GUS-overexpressing hairy root line grown under dark conditions (control line); G-L: GUS-overexpressing hairy root line grown under light conditions (control line); M-9-D; *AtMYB12*-overexpressing hairy root line grown under dark conditions (transgenic line); M-9-L: *AtMYB12*-overexpressing hairy root line grown under light conditions (transgenic line).

Analysis of metabolites from control and *AtMYB12* overexpression hairy root lines by HPLC and GC-TOFMS showed that in total 50 metabolites were identified (amines, carotenoids, amino acids, glucosinolates, phenolic acids, organic acids, sugar alcohols, and sugars) (Figures S3–S6). The heat map result showed that most of the metabolites were significantly higher in the transgenic line grown under light and dark conditions. The heat map was divided into two major clusters, namely cluster 1 and cluster 2 (Figure 5). Then cluster 1 was subdivided into clusters 1-1 and 1-2, whereas cluster 2 was also divided into clusters 2-1 and 2-2. Cluster 2 was further sub-grouped into two groups, namely 2-2a and 2-2b. Cluster 1 consisted of metabolites rich in the control hairy root lines. Cluster 1-1 consisted of metabolites rich in the control hairy root lines grown under both light and dark conditions, whereas cluster 1-2 consists of the metabolites that were significantly richer in the control hairy root lines grown under the light conditions. Cluster 2-1 comprises the metabolites abundant in the control and transgenic hairy root grown under dark conditions, whereas the 2-2 were separated based on the low level of metabolites present in the control hairy root lines. As mentioned above, cluster 2-2 was divided into 2-2a and 2-2b; cluster 2-2a consisted of metabolites richer in the transgenic hairy root culture lines grown under light and dark conditions and in the control hairy root cultures grown under lighted conditions. Cluster 2-2b comprises the metabolites abundant in the transgenic hairy root lines that grow in light and dark conditions.

In detail, the intermediate tricarboxylic (TCA) cycle products, such as pyruvic acid, succinic acid, and malic acid were significantly higher in the control hairy root cultures grown under the lighted conditions, whereas the glyceric acid was highest in the transgenic lines grown under the lighted conditions (Figure 5). Most of the sugars and sugar alcohols showed differential accumulation when the transgenic and control hairy root cultures were grown under light and dark conditions. The sugars, such as xylose, arabinose, galactose, glucose, and fructose, showed the highest accumulation in the control lines grown under dark conditions, whereas fructose, mannose, mannitol, and sucrose were significantly accumulated in the transgenic hairy root grown under dark conditions. The sugar alcohols, such as glycerol and inositol, showed the highest values in the control and transgenic hairy root cultures grown under dark conditions. This indicates that the dark conditions enhanced the sugar and sugar alcohol accumulation in the control and transgenic hairy root cultures. The individual amino acid levels also varied among the control and transgenic hairy roots cultures. The levels of valine, threonine, lysine, tryptophan, isoleucine, and leucine significantly increased in the transgenic hairy root cultures grown under the dark conditions followed by the control hairy root cultures grown under the light and dark conditions, whereas all the above amino acids significantly decreased in the transgenic hairy root cultures grown under the lighted conditions. The amino acids, such as serine, alanine, β-alanine, and tyrosine were significantly higher only in the transgenic hairy root cultures grown under light conditions. Significantly higher amounts of glycine, methionine, aspartic acid, and glutamine were found only in the transgenic hairy root cultures grown under light and dark conditions. The shikimate pathway derivatives, such as tyrosine, tryptophan, and phenylalanine, showed a differential accumulation; they were higher in the control hairy root cultures grown under lighted conditions, transgenic hairy root cultures grown under dark conditions, and control hairy root grown under lighted conditions, respectively. From this result, it is shown that most of the metabolites were higher in the transgenic hairy root grown cultures under light and dark conditions and were especially significantly higher in the transgenic hairy root cultures grown under light conditions.

**Figure 6:**
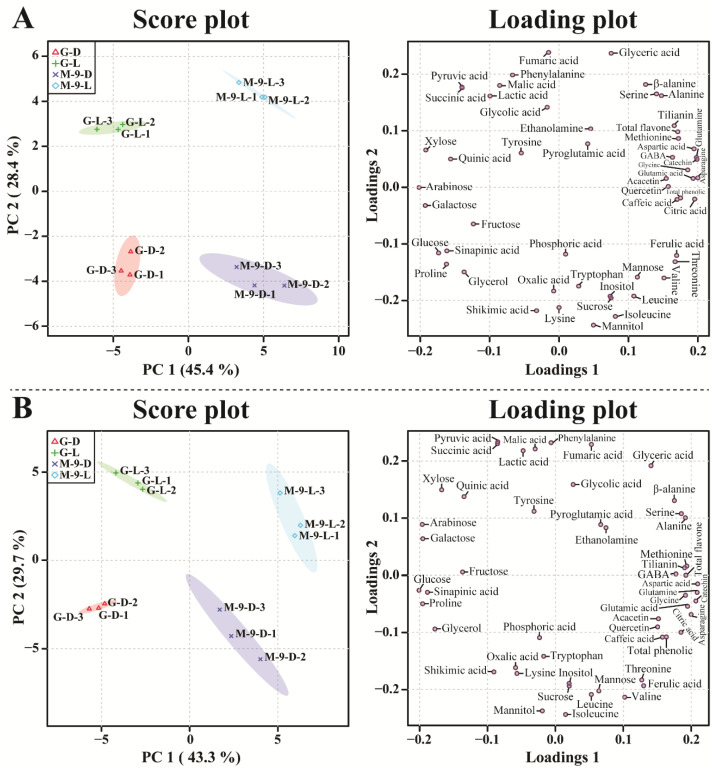
Score and loading plots of the PCA (**A**) and PLS-DA (**B**) models of the metabolites obtained in control and transgenic hairy root cultures of Korean mint grown under light and dark conditions. G-D: GUS-overexpressing hairy root line grown under dark conditions (control line); G-L: GUS-overexpressing hairy root line grown under light conditions (control line); M-9-D; *AtMYB12*-overexpressing hairy root line grown under dark conditions (transgenic line); M-9-L: *AtMYB12*-overexpressing hairy root line grown under light conditions (transgenic line).

**Figure 7:**
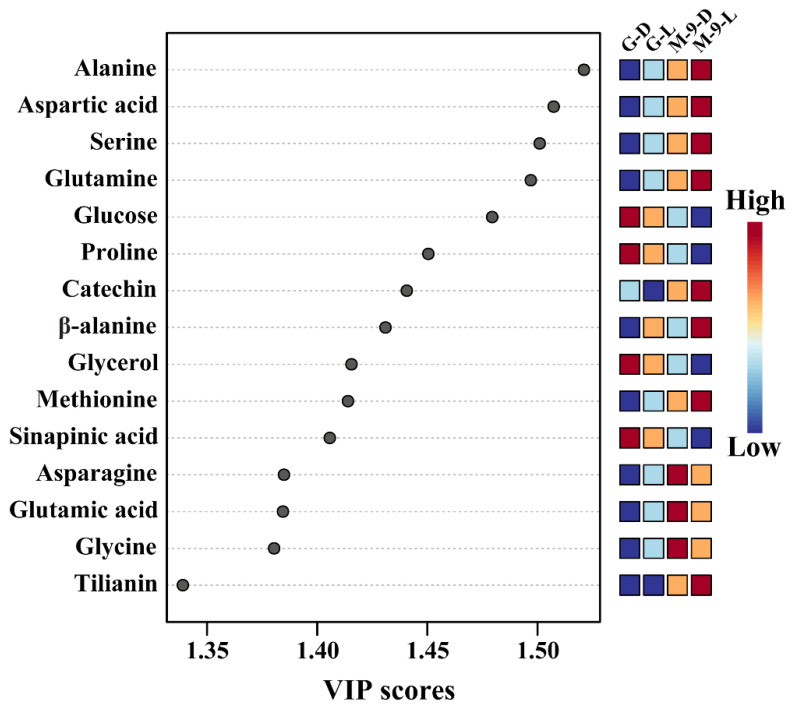
The main components, differentiating the control and transgenic Korean mint hairy root cultures grown under light and dark conditions based on the VIP scores obtained using the PLS-DA model. Colored boxes on the right indicate relative concentrations of the corresponding control and transgenic Korean mint hairy root cultures grown under light and dark conditions. The x-axis represents the VIP score, whereas the y-axis represents the top 15 important components that correspond to the control and transgenic Korean mint hairy root cultures grown under light and dark conditions and are organized in descending order of the VIP score. Red color indicates high and blue color indicates low. G-D: GUS-overexpressing hairy root line grown under dark conditions (control line); G-L: GUS-overexpressing hairy root line grown under light conditions (control line); M-9-D; *AtMYB12*-overexpressing hairy root line grown under dark conditions (transgenic line); M-9-L: *AtMYB12*-overexpressing hairy root line grown under light conditions (transgenic line).

PCA analysis was executed to examine the differences in the identified metabolites detected in the control and transgenic hairy root exposed to light and dark conditions. The PCA analysis results showed that the control and transgenic hairy root cultures grown under light and dark conditions were completely separated from each other, which resulted in the 45.4% (principal component 1 (PC1)) and 28.4% (PC2) variance with two-component analysis (Figure 6). This separation might be due to the amino acids, TCA cycle intermediates, sugars, and sugar alcohols. In the PCA analysis, asparagine, catechin, glutamine, citric acid, aspartic acid, glutamic acid, glycine, total phenolics, methionine, and total flavones had the eigenvector values of 0.20003, 0.19871, 0.19806, 0.19543, 0.19453, 0.1932, 0.18541, 0.17514, 0.17212, and 0.17108, respectively, and arabinose, galactose, xylose, glucose, proline, sinapinic acid, quinic acid, pyruvic acid, succinic acid, and glycerol had the eigenvector values of −0.2016, −0.19275, −0.19194, −0.17354, −0.1622, −0.1616, −0.15614, −0.13987, −0.13969, and −0.13674, respectively. The PLS-DA analysis results also showed complete separation from each other, which showed 43.3% and 29.7% of the variance with two-component analysis. This separation might be due to the subsequent metabolites; aspartic acid, glutamine, catechin, asparagine, glutamic acid, methionine, total flavone, glycine, alanine, and tilianin had eigenvector values of 0.20958, 0.20876, 0.20687, 0.19992, 0.19464, 0.19356, 0.19218, 0.1916, 0.19146, and 0.19037, respectively, and glucose, arabinose, proline, galactose, sinapinic acid, glycerol, xylose, fructose, quinic acid, shikimic acid, whose eigenvector values of −0.20091, −0.19611, −0.19556, −0.19535, −0.18851, −0.17748, −0.16752, −0.13676, −0.13461, and −0.090567, respectively. A clear separation was detected in the PCA and PLS-DA models and the metabolites, which showed a significant VIP value (>1): alanine, aspartic acid, serine, glutamine, glucose, proline, catechin, β-alanine, glycerol, methionine, sinapinic acid, asparagine, glutamic acid, glycine, and tilianin (Figure 7). This result supports the heatmap results, which showed that a significant difference between the primary and secondary metabolism in the control and transgenic plants exposed to light and dark conditions might be due to amino acids, TCA cycle intermediates, sugars, sugar alcohols, phenolic, and flavone compounds.

**Figure 8:**
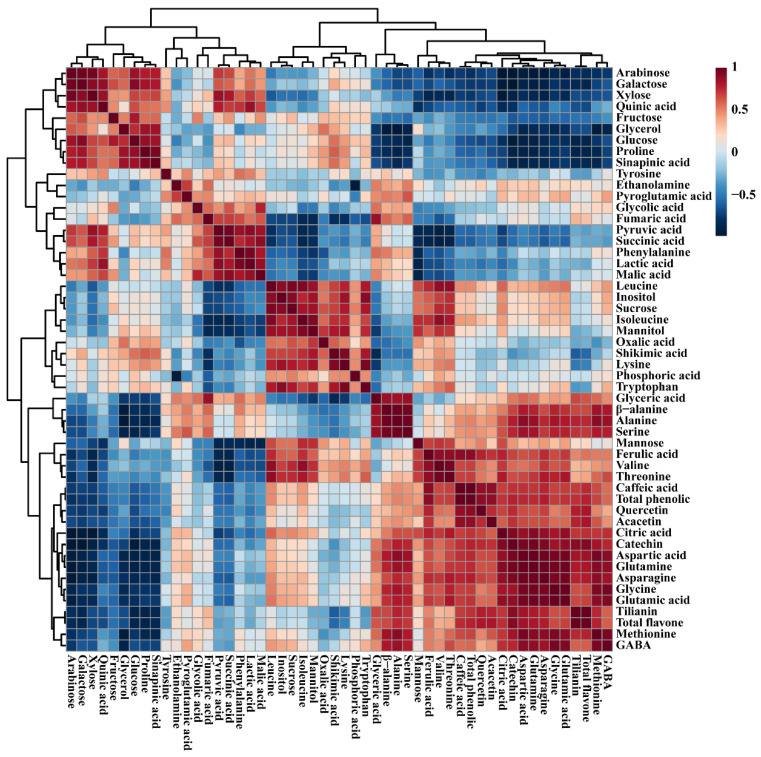
Relationships between the metabolites identified in the control and transgenic hairy root cultures of Korean mint grown under light and the dark are depicted in a correlation matrix; each coloured box shows Pearson’s correlation coefficient for a pair of compounds; the strength of the blue or red hue, as shown on the colour scale, denotes the correlation coefficient value for each coloured box.

The correlations between the identified metabolites in the control and transgenic hairy root cultures were examined using Pearson’s correlation (Figure 8). The shikimate pathway is the only well-known pathway for the biosynthesis of aromatic amino acids, such as phenylalanine, tryptophan, and tyrosine. The intermediate compound shikimic acid showed a positive correlation with tryptophan and tyrosine, whereas it showed a negative correlation with phenylalanine. In addition, shikimic acid showed a positive correlation with most sugars (fructose, glucose, sucrose, and mannose) and sugar alcohols (glycerol, inositol, and mannitol). Phenylalanine showed a positive correlation with most of the TCA cycle intermediates such as fumaric acid (*r* = 0.65654, *p* = 0.020392), malic acid (*r* = 0.81878, *p* = 0.001126), and succinic acid (*r* = 75701, *p* = 0.004362), whereas it showed a negative correlation with citric acid. Glutamic acid, including its by-products, such as glutamine, and pyroglutamic acid, showed a positive correlation with citric acid. In addition, except for proline and tyrosine, all other amino acids showed a positive correlation with citric acid, and among these, glutamine showed a strong relationship with citric acid (*r* = 0.91069, *p* = 0.0000). Furthermore, a significant strong positive correlation between glutamic acid and gamma-aminobutyric acid (GABA) (*r* = 0.92922, *p* = 0.0000) was also detected. Sucrose had a positive correlation with the total phenolic content and with all the identified individual phenolic compounds such as caffeic acid, ferulic acid, catechin, and quercetin, whereas in flavone compounds only acacetin showed a positive correlation. In addition, sucrose showed a positive correlation with GABA.

**Figure 9:**
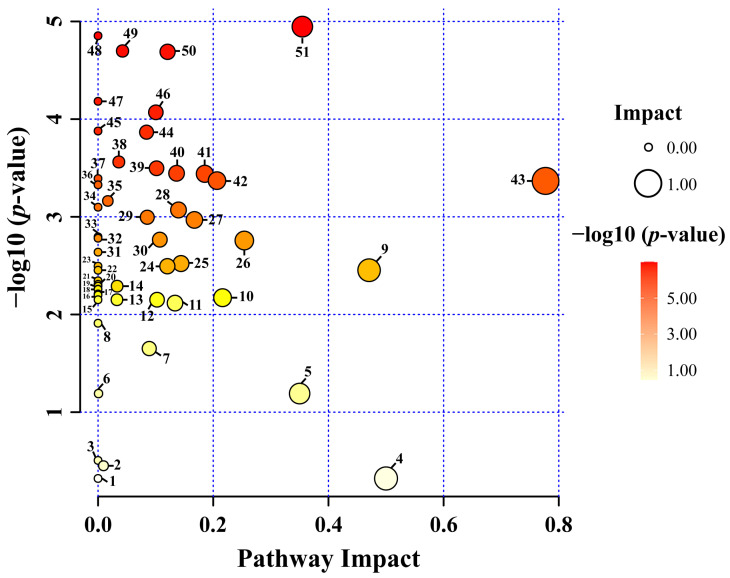
Identified metabolites and their pathway impact on control and transgenic hairy root of Korean mint grown under light and dark conditions. The x-axis represents the pathway impact, and the y-axis represents −log10 (*p*-value). Large sizes and dark colors represent major pathway enrichment and high pathway impact values, respectively. The numbers appearing in the figure correspond to the impacted pathways mentioned in the above paragraph.

Fifty-four pathways were identified in the control and transgenic hairy root lines. *Thale cress* Kyoto Encyclopaedia of Genes and Genomes (KEGG) were selected for the metabolic pathway analysis (Table S2). Among these, 30 pathways were discovered to be impacted in this study; 6 belong to the amino acid biosynthesis and metabolic pathways: alanine, aspartate, and glutamate metabolism; glycine, serine, and threonine metabolism; tyrosine metabolism, arginine and proline metabolism; phenylpropanoid biosynthesis; and arginine biosynthesis. In addition, glyoxylate and dicarboxylate metabolism, the Citrate cycle (TCA cycle), pyruvate metabolism, inositol phosphate metabolism, starch metabolism, and sucrose metabolism, and galactose metabolism, which belongs to the carbohydrate metabolism pathway, were also impacted. Butanoate metabolism, glutathione metabolism, isoquinoline alkaloid biosynthesis, and primary metabolic pathways were also impacted. However, metabolisms, such as thiamine metabolism, cyanoamino acid metabolism, pyrimidine metabolism, selenocompound metabolism, nitrogen metabolism, purine metabolism, and a few other metabolisms were not impacted. In detail, the identified metabolites and their pathway impact on control and transgenic hairy root of Korean mint grown under light and dark conditions are; (1) Ubiquinone and other terpenoid–quinone biosynthesis, (2) Glycerophospholipid metabolism, (3) Fatty acid degradation, (4) Isoquinoline alkaloid biosynthesis, (5) Flavone and flavonol biosynthesis, (6) Flavonoid biosynthesis, (7) Starch and sucrose metabolism, (8) Amino sugar and nucleotide sugar metabolism, (9) Phenylalanine metabolism, (10) Tyrosine metabolism, (11) Glutathione metabolism, (12) Inositol phosphate metabolism, (13) Phosphatidylinositol signaling system, (14) Sulfur metabolism, (15) Ascorbate and aldarate metabolism, (16) Nicotinate and nicotinamide metabolism, (17) Purine metabolism, (18) Nitrogen metabolism, (19) Propanoate metabolism, (20) Lysine degradation, (21) Valine, leucine and isoleucine degradation, (22) Tropane, piperidine and pyridine alkaloid biosynthesis, (23) Indole alkaloid biosynthesis, (24) Tryptophan metabolism, (25) Arginine and proline metabolism, (26) Beta-alanine metabolism, (27) Pyruvate metabolism, (28) Cysteine and methionine metabolism, (29) Arginine biosynthesis, (30) Valine, leucine and isoleucine biosynthesis, (31) Terpenoid backbone biosynthesis, (32) Selenocompound metabolism, (33) Pyrimidine metabolism, (34) Cyanoamino acid metabolism, (35) Glycerolipid metabolism, (36) Monobactam biosynthesis, (37) Thiamine metabolism, (38) Carbon fixation in photosynthetic organisms, (39) Phenylalanine, tyrosine and tryptophane biosynthesis, (40) Butanoate metabolism, (41) Citrate cycle (TCA cycle), (42) Glyoxylate and dicarboxylate metabolism, (43) Alanine, aspartate, and glutamate metabolism, (44) Pantothenate and CoA biosynthesis, (45) Glucosinolate biosynthesis, (46) Phenylpropanoid biosynthesis, (47) Aminoacyl-tRNA biosynthesis, (48) Lysine metabolism, (49) Galactose metabolism, (50) Glycolysis/Gluconeogenesis, and (51) Glycine, serine, and threonine metabolism (Figure 9). These findings imply that different nutraceutical pathways were impacted.

## 3. Discussion

The *MYB* transcription factors family is large and participates in a variety of physiological and biochemical processes, including environmental stress responses and modulation of secondary metabolism [19,43]. In addition, several studies reported that the *MYB* transcription family is a key regulator for phenylpropanoid synthesis [44]. Regarding the *MYB* family, *MYB12* was revealed as a flavonoid regulator in thale cress [24,32]. In tomatoes, *AtMYB12* positively regulated caffeoyl quinic acid and flavonol production [45,46]. Moreover, some *MYB* transcription factors also enhanced the phenylpropanoid accumulation in hairy roots, including *FtMYB1*, *FtMYB2*, *FtMYB3*, and *FtMYB*-like overexpression in Tartary buckwheat hairy root cultures [47], *AtMYB12* overexpression in *F. esculentum* hairy root [30], and *SbMYB12* overexpression in *Scutellaria baicalensis* (Chinese skullcap) hairy root cultures [48]. A similar result was obtained in this study, that *AtMYB12* positively regulated not only the phenolic compounds, such as caffeic acid, ferulic acid, catechin, and quercetin but also flavone compounds including acacetin and tilianin in the transgenic Korean mint hairy root line compared with the control line. In the present study, the expression level of phenylpropanoid pathway genes and the phenolic and flavone contents were positively correlated. Earlier studies reported that the expression pattern of the *PAL* gene corresponded to the accumulation pattern of phenolic compounds [49,50]. In addition, a few studies showed that *MYB12* induced the transcription of the *PAL* gene [30,32,45]. In our study, the higher transcription level of *ArPAL* genes was achieved in the transgenic lines, which led to a higher accumulation of caffeic acid and ferulic acid. Additionally, *AtMYB12* was performed as a regulator of *CHS* and *CHI* genes. Previously, several studies reported that there was a significant positive correlation between *MYB12* and phenylpropanoid pathway genes (*CHS* and *CHI*) to increase the flavonoid content in thale cress [24,26], *Malus hupehensis* (Chinese crab apple) [51], *Nicotiana tabacum* (tobacco), and tomato [45]. Similarly, in our study, *ArCHS* and *ArCHI* expression levels were significantly increased which led to the highest accumulation of phenolic and flavone content in Korean mint transgenic hairy root lines. Interestingly, the tilianin content was significantly increased in the transgenic hairy root compared to that of the control line. This might be due to the expression level of *ArCHS* and *ArCHI* being higher in the transgenic lines when compared to the control line. These results indicated that *AtMYB12* triggers the phenylpropanoid pathway genes which led to the highest accumulation of phenolic and flavone content in the transgenic hairy root line.

Among flavonoid biosynthesis regulators, light plays an important role in inducing the transcription of phenylpropanoid pathway genes [52]. The impact of light on flavonoid accumulation was reported in several plants such as thale cress [53], *Ligustrum vulgare* (common privet) [54], *Oryza sativa* (rice) [55], and common buckwheat [56]. The effect of light on *MYB* genes was reported in Petunia [57], *Malus domestica* (apple) [58], and Tartary buckwheat [59]. Moreover, some researchers indicated that under continuous dark incubation, there was a negative regulation of the *MYB12* gene, which led to a several-fold reduction in the expression of phenylpropanoid pathway genes and flavonoid compounds [60,61]. It was reported that in *Arabidopsis*, the combination of *MYB* transcription factors such as *MYB12*, *MYB111*, and *MYB11*, with the bZip family transcription factor HY5, were necessary for activating the expression of flavonoid structural genes, leading to an increase in the flavonoid content [62]. Additionally, HY5 is considered as light-dependent regulator for secondary metabolite production in plants [63] and *MYB12* was also positively regulated by light [40]. These two factors are implicated in activating phenylpropanoid pathway genes, especially *CHS* genes [62]. In this research, phenolic and flavone contents in the transgenic hairy root line were not increased significantly in dark treatment, whereas, in the continuous light treatment, the phenolic and flavone contents were significantly increased in the transgenic hairy root cultures compared to that of the control hairy root line. Therefore, it was shown that the *AtMYB12* transcriptional factor is necessary for the activation of phenylpropanoid pathway genes, leading to enhance the accumulation of phenolic and flavone compounds in Korean mint transgenic hairy root cultures.

Sucrose is easily transferred across membranes, and it can be easily digested by the cells using a sucrose transporter, hence it has been employed as one of the important carbohydrate sources for plant tissue cultures [64]. In this study, the sucrose level was high in the control and transgenic plants. This could be because leaves are sugar sources that produce sucrose and transmit it to the sink organs, whereas bulbs and roots are sugar sinks that store sucrose. A previous study reported that bulbs and roots contain a higher sucrose concentration than leaves. A similar result was reported by Du Toit et al. [65], who reported that *Lachenalia* cv. Ronina roots and bulbs served as the plant’s primary organs for storing sucrose, while its inflorescence and leaves served as its primary sources of sugar. In addition, it was noted that when sucrose is produced in leaves as a source of sugar, it is exported to other plant parts via the phloem, thus leaves do not possess it for long [66]. A positive relationship between sucrose and phenolic and flavone compounds was detected. This result was consistent with several studies that claim sucrose is essential for the biosynthesis of secondary metabolites, such as alkaloids [67], anthocyanin [68], phenolics [69], and triterpenoids [70]. Previous studies reported that sucrose enhances the accumulation of phenolic compounds in *Cucumis anguria* (maroon cucumber) [71], *Aster scaber* (shirayamagiku) [72], and Tartary buckwheat [73] hairy root cultures. In addition, another study reported that sucrose increases the withaferin A and withanone content in the *Withania somnifera* (Ashwagandha) hairy root cultures [74]. Similarly, sucrose increased the accumulation of flavonol contents in the hairy root culture of Chinese skullcap [75]. In addition, sucrose enhances the accumulation of paclitaxel content in the cell suspension culture of *Taxus chinensis* (the Chinese yew) [76] and increases the yield of anthraquinone, flavonoids, and phenolics in *Morinda citrifolia* (Indian mulberry) adventitious root suspension cultures [69]. Moreover, it has been reported that sucrose increased the withaferin A content in Ashwagandha adventitious root cultures [74], and that transgenic hairy root cultures *showed* the highest accumulation of several phenolic and flavonoid compounds in several plants, such as Sphagneticola calendulacea (Chinese wedelia) [77], *Brassica rapa (wild turnip)* [78], *Dracocephalum moldavica* (Moldavian dragonhead) [79], and *Momordica dioica* (spiny gourd) [80]. The shikimate pathway leads to the formation of tryptophan, which is used as a precursor for indolic glucosinolate metabolism, whereas phenylalanine is a substrate for phenylpropanoid metabolism [81]. In this study, the highest shikimic acid content was in the transgenic hairy root cultures grown under lighted conditions, thus leading to a higher accumulation of total phenolic content than that of the control hairy root cultures. Similarly, the GABA content was higher in the transgenic hairy root culture than in the control hairy root cultures, and it showed a strong positive correlation with glutamic acid. Goyal et al., [82] reported that plant secondary metabolites, such as GABA, flavonoids, and polyphenols, were synthesised from the primary carbon metabolism intermediates through the phenylpropanoic acid and glutamic acid pathways. This led to higher accumulations of phenolic and flavone contents in the transgenic hairy root cultures than that in the control hairy root cultures.

## 4. Materials and Methods

### 4.1. Plant Materials and Seed Germination

The Korean mint seeds were purchased from the Aram company (Seoul, Republic of Korea) and were soaked in distilled water for 3 h and quickly dipped in 70% ethanol *(v/v)* for 1 min. Then, seeds were immersed for 10 min in 4% (*v*/*v*) NaClO containing a few drops of Tween 20, followed by rinsing with autoclaved distilled water for five times and dried with sterilized papers. Then, the seeds were germinated in half-strength autoclaved MS medium with 0.8% plant agar (pH 5.8) and incubated in a growth chamber under a 16:8 light-dark photoperiod for 2 weeks. The resulting Korean mint plant seedlings were used for hairy root induction.

### 4.2. Hairy Root Induction

Transformation protocol was performed following the protocol described by Li et al. [47] with slight modification. *A. rhizogenes* R1000 strains harbouring pB7FWG2 with *AtMYB12* gene, and control line pB7FWG2 with *β*-glucuronidase overexpression vector (GUS), were grown in a 30 mL of LB liquid medium containing 1% spectinomycin (50 mg/mL) at 27 °C for 1 day with continuous shaking at 180 rpm. After centrifugation, the supernatant was removed, the bacteria pellet was resuspended with half-strength SH liquid medium, and the cell suspension was adjusted to A_600_ = 0.6. Young leaves and stems from the Korean mint seedlings were excised, and the explants were cut into 1 × 1 cm^2^ pieces and then immersed in the *A. rhizogenes* suspension for 20 min, and then dried with autoclaved tissue paper. The infected explants were incubated in a ½ strength SH agar medium at 25 °C under dark conditions. After 2 days of co-cultivation, these infected explants were subculture with half-strength SH medium containing cefotaxime (500 mg/mL) antibiotic. A week later, these explants were transferred to solidified half-strength SH selection medium containing cefotaxime (500 mg/mL) and phosphinothricin (2 mg/mL) and incubated at 25 °C in the dark. When the hairy roots appeared at infectious sites, each of them was cut and transferred to the above medium containing antibiotics and incubated at 25 °C for 4 weeks in a growth chamber under dark conditions. Subsequently, sub-cultured hairy roots were collected, and 6 g of each hairy root line was transferred to 30 mL of half-strength SH liquid medium and incubated in a growth chamber at 25 °C under continuous shaking (100 rpm). Two different conditions were followed: (1) continuous darkness and (2) continuous exposure to cool white fluorescent lights with a flux rate of 35 µmol m^−2^ s^−1^. After 30 days of culture, hairy roots were harvested and frozen with liquid nitrogen. Small amounts of sample powders were used for RNA extraction and the remaining were freeze-dried for primary and secondary metabolite analysis. All the analyses were done in triplicate.

### 4.3. RNA Extraction and cDNA Synthesis

Total RNA was extracted from the control and 15 *AtMYB12*-overexpressing hairy root lines of Korean mint using the CTAB method, and RNA purification was done using RNeasy Plant Mini Kit (Qiagen, Valencia, CA, USA). These RNA were converted to cDNA by using the ReverTra Ace-R Kit (Toyobo Co., Ltd., Osaka, Japan) according to the manufacturer’s procedure. The synthesised cDNA was diluted 20 times as a template for qRT-PCR.

### 4.4. Quantitative Real-Time PCR Analysis

The expression levels of the control and *AtMYB12*-overexpressing hairy root lines were analysed by using qRT-PCR. The *AtMYB12* gene-specific primers were used for qRT-PCR analysis [30]. The housekeeping gene, *actin*, was used as an internal control [83]. The PCR conditions were as follows: pre-denaturation step at 95 °C for 15 min, followed by denaturation at 95 °C for 15 s, repeated 40 cycles, annealing at 58 °C for 15 s, and elongation at 72 °C for 20 s. The highest *AtMYB12* expression hairy root line was selected and the phenylpropanoid pathway genes (*ArPAL*, *ArC4H*, *Ar4CL*, *ArCHS*, *ArCHI*) expression was further analysed by using the gene-specific primers [83]. The PCR condition started with a pre-denaturation step at 95 °C for 15 min, followed by denaturation at 95 °C for 15 s, repeated 40 cycles, annealing at 59 °C for 15 s, and elongation at 72 °C for 20 s. For each reaction, three replicates were used.

### 4.5. Extraction and HPLC Analysis of Phenolic and Flavones Compounds

Phenolic compounds were extracted and analysed according to the protocol described by Park et al. [84]. For flavone compound extraction, 100 mg of powder control and transgenic hairy root grown under light and dark conditions samples were collected, 1.2 mL of 80% methanol (*v/v*) were added, and the samples were vortexed for 1 min. After that, these mixtures were sonicated for 1 h, followed by centrifugation at 1200 rpm at 4 °C for 20 min. Next, the supernatant of each sample was taken and filtered with a 0.45-µm PVDF filter into a vial for HPLC (NS-4000, Futecs, Daejeon, Republic of Korea) analysis. The analysis condition was similar to that of the protocol described by Park et al. [33]. The mobile phase consisted of absolute methanol (solvent A) and water containing 0.2% (*v/v*) acetic acid (solvent B). Compounds were detected at 340-nm wavelengths. The compounds were identified by comparing the retention time of the standards and spiked tests and using the calibration curves for determining the quantification of the compounds in the samples.

### 4.6. GC-TOFMS Analysis

Hydrophilic compound extraction and analysis were carried out following the protocol reported by Sathasivam et al. [85]. To 10 mg of well-crushed samples, 1mL of methyl alcohol/chloroform/water (2.5:1:1, *v*/*v*/*v*) was added. As an internal standard, 60 μL of ribitol (0.2 g/L) was added, mixed gently, and centrifuged at 160,000× *g* for 5 min. Then, the methanol/water phase was dried with a centrifugal concentrator (CC-105, TOMY, Tokyo, Japan) for 3 h, followed by freeze-drying at −80 °C for 16 h. Then, 80 μL of methoxyamine hydrochloride in pyridine (20 mg/mL) was mixed with samples for methoxime derivatisation followed by adding 80 μL of N-methyl-N-(trimethylsilyl)trifluoroacetamide for trimethylsilyl etherification. For analysis, an Agilent 7890A GC (Agilent Technologies, Santa Clara, CA, USA) connected with a Pegasus TOF-MS (Leco, St. Joseph, MI, USA) was utilised. Hydrophilic compounds were separated using an Rtx-5MS column (30 m × 0.25 mm, 0.25-μm i.d. film thickness; Restek, Bellefonte, PA, USA). The relative ratio of the peak area of each compound to the peak area of the internal standard was quantified based on the selected ions (Table S3). The GC-TOFMS analysis was performed as described in a previous study by Sathasivam et al. [85].

### 4.7. Statistical Analysis

The data were calculated and evaluated according to the Student’s *t*-test with a significance level of 0.05 on GraphPad Prism 9 software. The PCA and PLS-DA, correlation analysis, HCA, heat map, pathway impact, and variable importance in projection (VIP) of the 50 identified metabolites from the control and transgenic hairy root cultures grown under light and dark conditions were performed using MetaboAnalyst 5.0 (http://www.metaboanalyst.ca/, accessed on 17 February 2023) with autoscaling.

## 5. Conclusions

To our knowledge, this is the first report to enhance the primary and secondary metabolites in the hairy root cultures of Korean mint by overexpressing the Tatary buckwheat transcription factor *MYB12*. The results showed that the *AtMYB12* transcription factor triggered the phenylpropanoid biosynthesis pathway genes, which led to the highest accumulations of primary and secondary metabolites in the transgenic hairy root lines compared to the control hairy root culture lines when grown under the lighted conditions. Therefore, the *AtMYB12* transcription factor could be light-specific, and it plays a significant role in both primary and secondary metabolism in Korean mint. This study showed that Korean mint can be a suitable plant source for targeted metabolic engineering application with the aim of high production of specific medicinally important metabolites.

## Data Availability

Not applicable.

## Supplementary Materials

The following supporting information can be downloaded at: https://www.mdpi.com/article/10.3390/life13041042/s1, Figure S1: Phenylpropanoid biosynthetic pathway represented in Korean mint hairy roots cultures with the overexpression of the *AtMYB12* gene after light and dark treatments, respectively; Figure S2: The relative gene expression levels of the *AtMYB12* gene in the transgenic hairy root cultures of Korean mint grown under dark conditions; Figure S3: GC-TOFMS hydrophilic compounds chromatogram obtained from Korean mint control hairy root cultures grown in dark conditions; Figure S4: GC-TOFMS hydrophilic compounds chromatogram obtained from Korean mint transgenic hairy root cultures grown in dark conditions; Figure S5: GC-TOFMS hydrophilic compounds chromatogram obtained from Korean mint control hairy root cultures grown in light conditions; Figure S6: GC-TOFMS hydrophilic compounds chromatogram obtained from Korean mint transgenic hairy root cultures grown in light conditions; Table S1: Total phenolics content of transgenics (M-3, M-4, M-9) and control (G) hairy root line of Korean mint; Table S2: Metabolic pathways where the identified metabolites are involved in control and transgenic hairy root of Korean mint grown under light and dark conditions; Table S3: Retention times (RTs), relative retention times (RRTs) and quantification ion data of hydrophilic compounds in control and transgenic hairy root of Korean mint grown under light and dark conditions.
