# Supplementary material for: Impact of Light and Dark Treatment on Phenylpropanoid Pathway Genes, Primary and Secondary Metabolites in Agastache rugosa Transgenic Hairy Root Cultures by Overexpressing Arabidopsis Transcription Factor AtMYB12
Source: Life (Basel). 2023 Apr 19;13(4):1042. doi: 10.3390/life13041042 (PMC10142052; doi:10.3390/life13041042)
Supplement: Supplementary file 1 [file life-13-01042-s001.zip › Figures S1–S6.pdf]

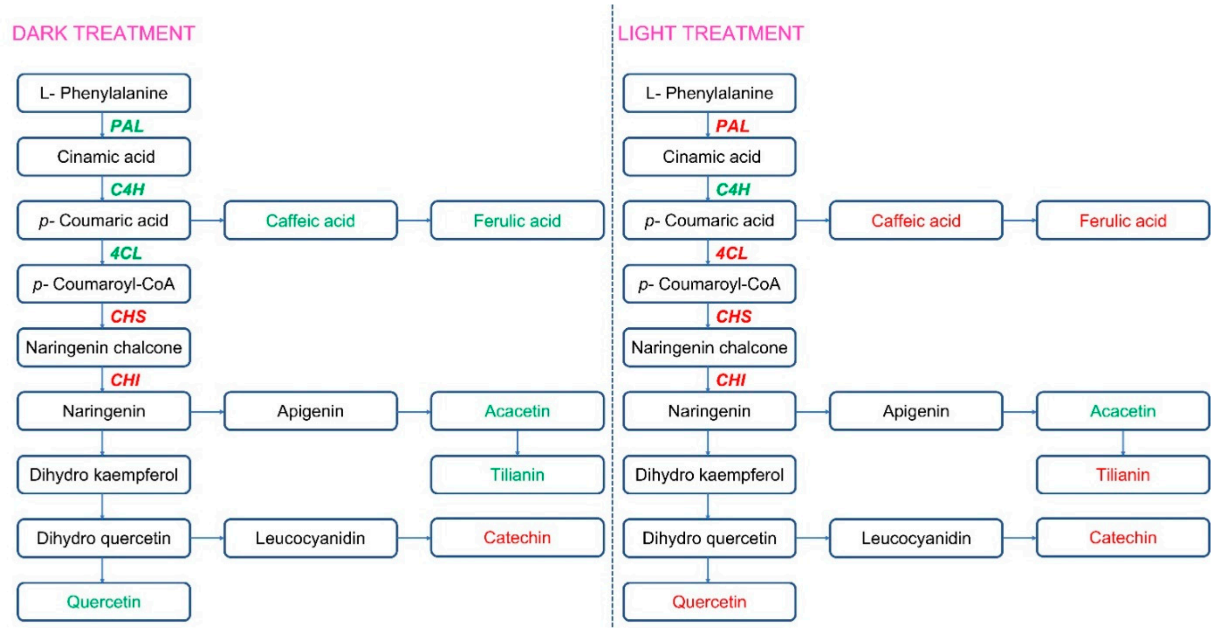


**Figure S1:** Phenylpropanoid biosynthetic pathway represented in Korean mint hairy roots cultures with the overexpression of the *AtMYB12* gene after light and dark treatments, respectively; the red text indicates a significant increase in phenolic and flavone compounds ($p < 0.05$) of transgenic hairy root lines cultured under light and dark conditions; the green text indicates phenolic and flavone compounds that failed to show significant differences; bolded italicized red text illustrates the significant up regulation of phenylpropanoid biosynthetic genes in transgenic hairy root lines exposed to light and dark treatments ($p < 0.05$), whereas the bold italicized green text shows no significant differences in the comparison of control and transgenic lines grown under dark and light conditions.

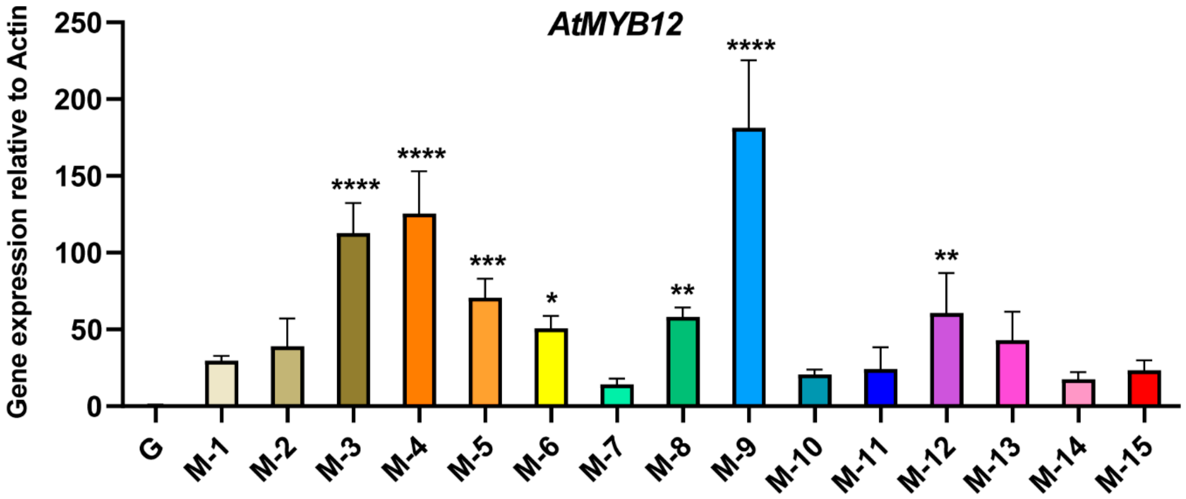


**Figure S2:** The relative gene expression levels of the *AtMYB12* gene in the transgenic hairy root cultures of Korean mint grown under dark conditions; asterisks describe statistical significance (* $p < 0.05$, ** $p < 0.01$, *** $p < 0.001$, **** $p < 0.0001$); control and *AtMYB12*-overexpressing hairy root lines are denoted as G and M, respectively.

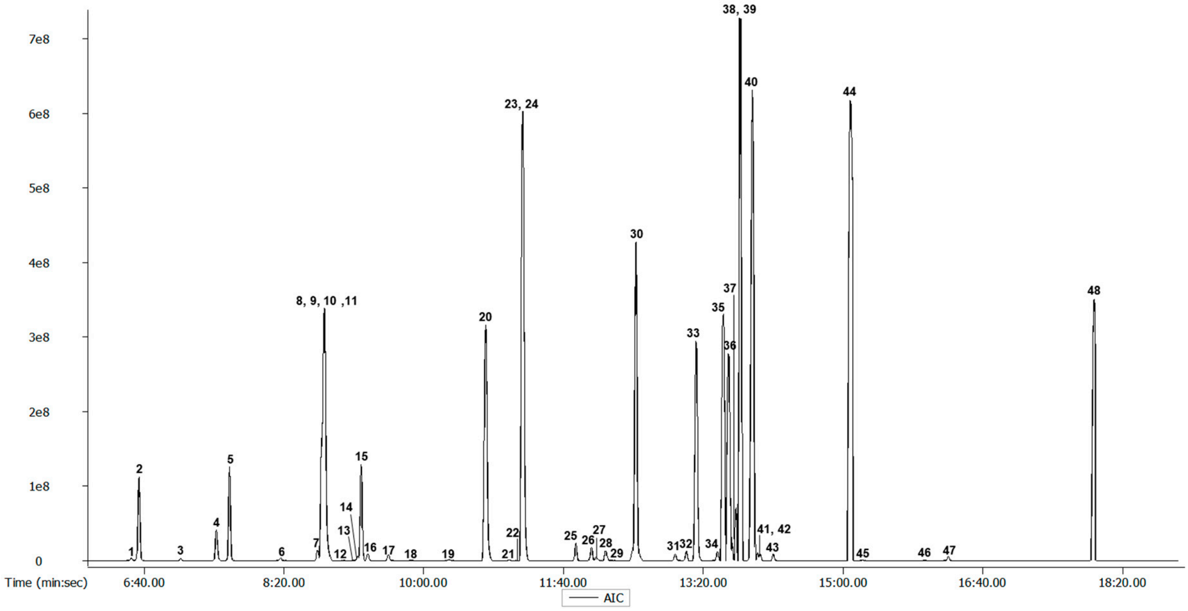


**Figure S3:** GC-TOFMS hydrophilic compounds chromatogram obtained from Korean mint control hairy root cultures grown in dark conditions. Peak: 1, Pyruvic acid; 2, Lactic Acid; 3, Alanine; 4, Oxalic acid; 5, Glycolic acid; 6, Valine; 7, Serine; 8, Ethanolamine; 9, Phosphoric acid; 10, Glycerol; 11, Leucine; 12, Isoleucine; 13, Proline; 14, Glycine; 15, Succinic acid; 16, Glyceric acid; 17, Fumaric acid; 18, Threonine; 19, β-Alanine; 20, Malic acid; 21, Aspartic acid; 22, Methionine; 23, Pyroglutamic acid; 24, 4-Aminobutyric acid (GABA); 25, Glutamic acid; 26, Phenylalanine; 27, Xylose; 28, Arabinose; 29, Asparagine; 30, Ribitol (internal standard); 31, Glutamine; 32, Shikimic acid; 33, Citric acid; 34, Quinic acid; 35, Fructose-1; 36, Fructose-2; 37, Mannose; 38, Galactose; 39, Glucose-1; 40, Glucose-2; 41, Mannitol; 42, Lysine; 43, Tyrosine; 44, Inositol; 45, Ferulic acid; 46, Tryptophan; 47, Sinapinic acid; 48, Sucrose.

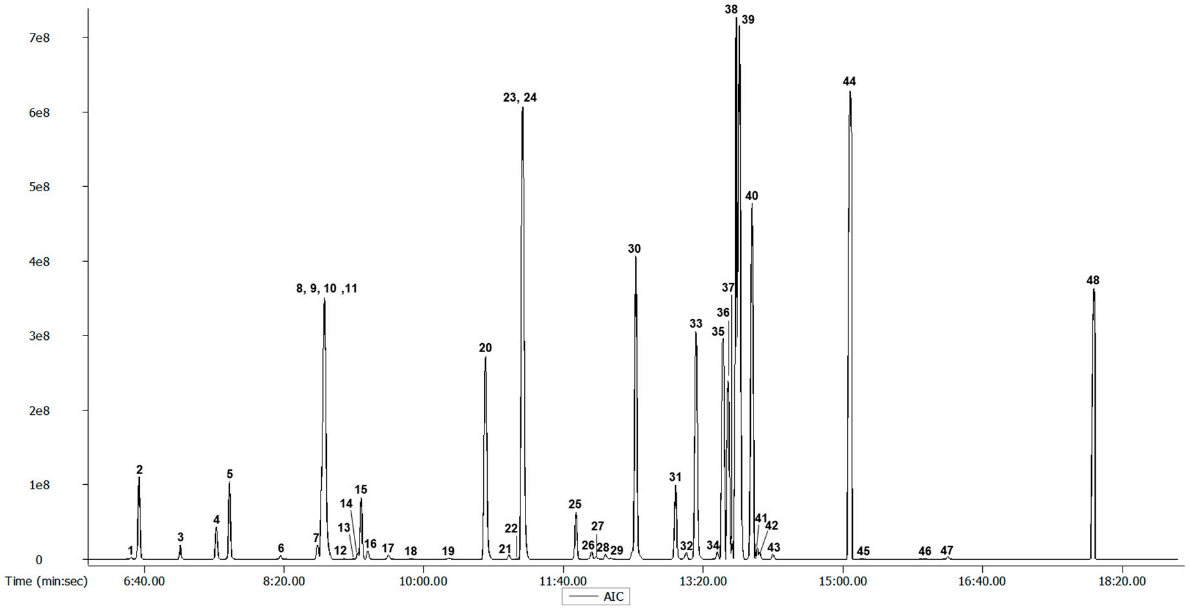


**Figure S4.** GC-TOFMS hydrophilic compounds chromatogram obtained from Korean mint transgenic hairy root cultures grown in dark conditions. Peak: 1, Pyruvic acid; 2, Lactic Acid; 3, Alanine; 4, Oxalic acid; 5, Glycolic acid; 6, Valine; 7, Serine; 8, Ethanolamine; 9, Phosphoric acid; 10, Glycerol; 11, Leucine; 12, Isoleucine; 13, Proline; 14, Glycine; 15, Succinic acid; 16, Glyceric acid; 17, Fumaric acid; 18, Threonine; 19, β-Alanine; 20, Malic acid; 21, Aspartic acid; 22, Methionine; 23, Pyroglutamic acid; 24, 4-Aminobutyric acid (GABA); 25, Glutamic acid; 26, Phenylalanine; 27, Xylose; 28, Arabinose; 29, Asparagine; 30, Ribitol (internal standard); 31, Glutamine; 32, Shikimic acid; 33, Citric acid; 34, Quinic acid; 35, Fructose-1; 36, Fructose-2; 37, Mannose; 38, Galactose; 39, Glucose-1; 40, Glucose-2; 41, Mannitol; 42, Lysine; 43, Tyrosine; 44, Inositol; 45, Ferulic acid; 46, Tryptophan; 47, Sinapinic acid; 48, Sucrose.

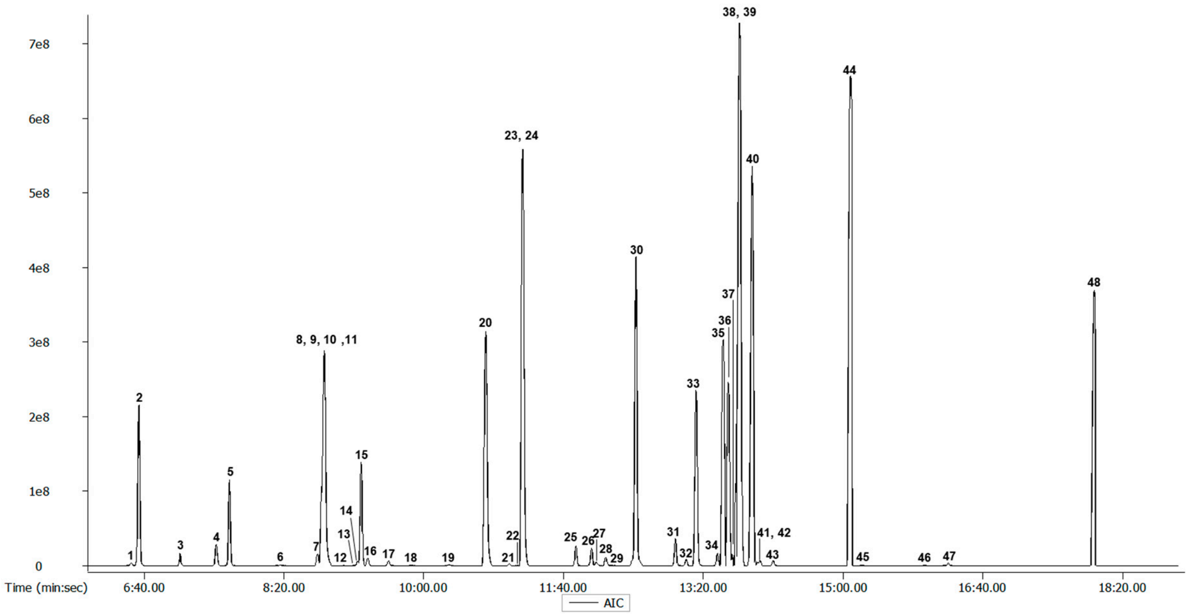


**Figure S5.** GC-TOFMS hydrophilic compounds chromatogram obtained from Korean mint control hairy root cultures grown in light conditions. Peak: 1, Pyruvic acid; 2, Lactic Acid; 3, Alanine; 4, Oxalic acid; 5, Glycolic acid; 6, Valine; 7, Serine; 8, Ethanolamine; 9, Phosphoric acid; 10, Glycerol; 11, Leucine; 12, Isoleucine; 13, Proline; 14, Glycine; 15, Succinic acid; 16, Glyceric acid; 17, Fumaric acid; 18, Threonine; 19, β-Alanine; 20, Malic acid; 21, Aspartic acid; 22, Methionine; 23, Pyroglutamic acid; 24, 4-Aminobutyric acid (GABA); 25, Glutamic acid; 26, Phenylalanine; 27, Xylose; 28, Arabinose; 29, Asparagine; 30, Ribitol (internal standard); 31, Glutamine; 32, Shikimic acid; 33, Citric acid; 34, Quinic acid; 35, Fructose-1; 36, Fructose-2; 37, Mannose; 38, Galactose; 39, Glucose-1; 40, Glucose-2; 41, Mannitol; 42, Lysine; 43, Tyrosine; 44, Inositol; 45, Ferulic acid; 46, Tryptophan; 47, Sinapinic acid; 48, Sucrose.

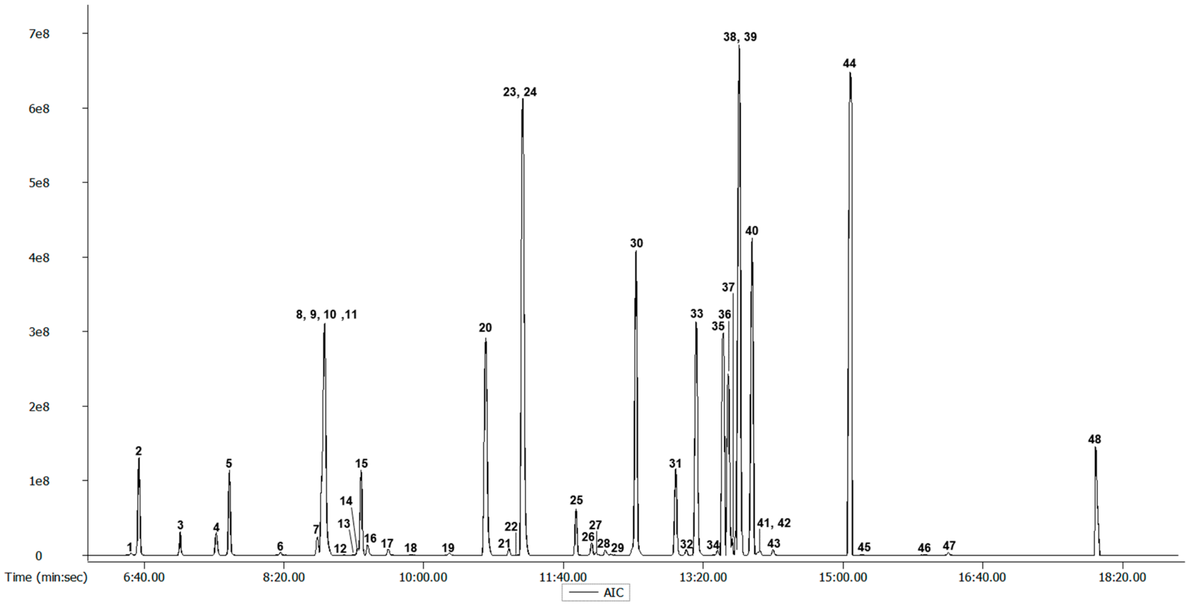


**Figure S6.** GC-TOFMS hydrophilic compounds chromatogram obtained from Korean mint transgenic hairy root cultures grown in light conditions. Peak: 1, Pyruvic acid; 2, Lactic Acid; 3, Alanine; 4, Oxalic acid; 5, Glycolic acid; 6, Valine; 7, Serine; 8, Ethanolamine; 9, Phosphoric acid; 10, Glycerol; 11, Leucine; 12, Isoleucine; 13, Proline; 14, Glycine; 15, Succinic acid; 16, Glyceric acid; 17, Fumaric acid; 18, Threonine; 19, β-Alanine; 20, Malic acid; 21, Aspartic acid; 22, Methionine; 23, Pyroglutamic acid; 24, 4-Aminobutyric acid (GABA); 25, Glutamic acid; 26, Phenylalanine; 27, Xylose; 28, Arabinose; 29, Asparagine; 30, Ribitol (internal standard); 31, Glutamine; 32, Shikimic acid; 33, Citric acid; 34, Quinic acid; 35, Fructose-1; 36, Fructose-2; 37, Mannose; 38, Galactose; 39, Glucose-1; 40, Glucose-2; 41, Mannitol; 42, Lysine; 43, Tyrosine; 44, Inositol; 45, Ferulic acid; 46, Tryptophan; 47, Sinapinic acid; 48, Sucrose.
