# Supplementary material for: Impact of Light and Dark Treatment on Phenylpropanoid Pathway Genes, Primary and Secondary Metabolites in Agastache rugosa Transgenic Hairy Root Cultures by Overexpressing Arabidopsis Transcription Factor AtMYB12
Source: Life (Basel). 2023 Apr 19;13(4):1042. doi: 10.3390/life13041042 (PMC10142052; doi:10.3390/life13041042)
Supplement: Supplementary file 1 [file life-13-01042-s001.zip › Tables S1 & S3.pdf]

Table S1. Total phenolics content of transgenics (M-3, M-4, M-9) and control (G) hairy root line of Korean mint.

Samples	Total phenolics (μg/g dry weight)
G	957.5 ± 52.7c
M-3	1005.5 ± 29.1c
M-4	1108.8 ± 7.3b
M-9	1203 ± 11.5a

Note: Control and *AtMYB12*-overexpressing hairy root lines were denoted as G and M, respectively. Mean values denoted with different letters are significantly different (p < 0.05, ANOVA, Duncan's multiple range test).

Table S3. Retention times (RTs), relative retention times (RRTs) and quantification ion data of hydrophilic compounds in control and transgenic hairy root of Korean mint grown under light and dark conditions.

S. No.	Compound	RT[a]	RRT[b]	Quantifiation ion[c]
1	Pyruvic acid	06:30.9	0.520	174
2	Lactic acid	06:36.3	0.527	117
3	Alanine	07:06.1	0.567	116
4	Oxalic acid	07:31.6	0.601	147
5	Glycolic acid	07:41.0	0.613	205
6	Valine	08:17.6	0.662	144
7	Serine	08:44.0	0.697	116
8	Glycerol	08:46.9	0.701	147
9	Phosphoric acid	08:48.9	0.704	299
10	Ethanolamine	08:49.0	0.704	174
11	Leucine	08:50.0	0.705	158
12	Isoleucine	09:03.1	0.722	158
13	Proline	09:10.4	0.732	142
14	Glycine	09:12.8	0.735	174
15	Succinic acid	09:15.3	0.739	147
16	Glyceric acid	09:20.0	0.745	147
17	Fumaric acid	09:34.8	0.765	245
18	Threonine	09:51.2	0.786	291
19	beta-Alanine	10:18.3	0.822	174
20	Malic acid	10:44.3	0.857	147
21	Aspartic acid	11:01.0	0.879	100
22	Methionine	11:06.6	0.887	176
23	Pyroglutamic acid	11:10.6	0.892	156
24	GABA	11:10.8	0.892	304
25	Glutamic acid	11:48.8	0.943	246
26	Phenylalanine	12:00.0	0.958	218
27	Xylose	12:03.3	0.962	103
28	Arabinose	12:10.1	0.971	103
29	Asparagine	12:13.7	0.976	116
30	Ribitol	12:31.8	1.000	217
31	Glutamine	13:00.0	1.038	156
32	Shikimic acid	13:07.8	1.048	204
33	Citric acid	13:14.9	1.057	273
34	Quinic acid	13:30.1	1.078	345
35	Fructose1	13:34.2	1.083	103
36	Fructose2	13:38.0	1.088	103
37	Mannose	13:40.6	1.092	319
38	Galactose	13:43.3	1.095	319
39	Glucose1	13:46.9	1.100	319
40	Glucose2	13:55.1	1.111	319
41	Mannitol	13:58.9	1.116	319
42	Lysine	14:00.7	1.118	174
43	Tyrosine	14:10.2	1.131	218

44	Inositol	15:05.2	1.204	305
45	Ferulic acid	15:13.8	1.215	338
46	Tryptophan	15:58.5	1.275	202
47	Sinapinic acid	16:15.3	1.297	338
48	Sucrose	17:59.6	1.436	217
